# Development of an Enzyme-Linked Immunosorbent Assay Method for the Detection of Rhein in *Rheum officinale*

**DOI:** 10.1155/2020/4294826

**Published:** 2020-03-16

**Authors:** Min Chen, Tie-Gui Nan, Jie Xin, Li Cui, Bo Zhang, Xiao Wang, Bao-Min Wang

**Affiliations:** ^1^School of Pharmacy, Linyi University, Linyi 276000, China; ^2^State Key Laboratory Breeding Base of Dao-di Herbs, National Resource Center for Chinese Materia Medica, China Academy of Chinese Medical Sciences, Beijing 100700, China; ^3^Key Laboratory for Applied Technology of Sophisticated Analytical Instruments of Shandong Province, Shandong Analysis and Test Center, Qilu University of Technology (Shandong Academy of Sciences), Jinan 250014, China; ^4^College of Agronomy and Biotechnology, China Agricultural University, Beijing 100193, China

## Abstract

Rhein is an important quality-control marker of *Rheum officinale*. The aim of this study was to develop an indirect competitive enzyme-linked immunosorbent assay (icELISA) for rhein detection, which acts as a powerful tool for quality control and proper usage of *Rheum officinale*. First, a specific and sensitive monoclonal antibody (mAb) against rhein was produced from a stable hybridoma cell line, 1F8, generated by the fusion of mouse myeloma sp2/0 with spleen cells obtained from a Bal b/c mouse immunized with rhein-BSA. Then, an icELISA method was developed with an IC_50_ value and working range of 0.05 *μ*g L^−1^ and 0.02–0.11 *μ*g L^−1^, respectively. The icELISA revealed high assay specificity, since it only had a relatively high cross reactivity with aloe-emodin (27%) and almost no cross reactivity with any other anthraquinones (<1%). When spiked with 0.2–2 mg kg^−1^ of rhein, the recoveries ranged from 84.19% to 102.90%. Finally, icELISA was used to detect rhein contents of *Rheum officinale* collected from different regions, and the results corresponded well with those of HPLC. Overall, the developed icELISA with high specificity and sensitivity provided a rapid and simple method for rhein detection, and it may be a powerful tool for quality control and proper usage of *Rheum officinale*.

## 1. Introduction

*Rheum officinale*, one of the oldest and best-known traditional Chinese medicines, has been used as a remedy for constipation and fever for thousands of years and is officially listed in many pharmacopoeias, such as China Pharmacopoeia, British Pharmacopoeia, European Pharmacopoeia, Korean Pharmacopoeia, and Japan Pharmacopoeia [1–5]. Rhein (4,5-dihydroxyanthraquinone-2-carboxylic acid), an important quality-control marker of rheum officinale, is the primary active compound that is absorbed by the body from ingestion of the rheum officinale extract [6, 7]. Rhein has been shown to exhibit a wide range of pharmacological activities, which include antitumor, anti-inflammatory, antiallergic, antiatherosclerosis, antidiabetic, antimicrobial, antibacterial, antifungal, antiviral, antigenotoxic, hepatoprotective, and nephroprotective properties [8, 9]. However, rhein is hepatotoxic [10], and one of its metabolites, rhein acyl glucuronide, is cytotoxic [11]. Thus, establishing an efficient and rapid method for determination of rhein concentrations is important for the quality control and proper usage of *Rheum officinale*.

At present, a variety of analytical methods, including high-performance thin-layer chromatography (HPTLC) [12], high-performance liquid chromatography (HPLC) [13], ultra-performance liquid chromatography (UPLC) [14], capillary electrophoresis (CE) [15–17], and electrochemical methods [18], have been used for quantitative detection of rhein in *Rheum officinale* and other herbs. However, the sensitivity of HPTLC is low, while other existing methods have relatively limited efficiency and longer analysis times. Compared to the methods mentioned above, immunoassays are rapid, sensitive, and only require small quantities of test materials and involve simple pretreatments. Moreover, immunoassays are inexpensive, ecofriendly, and amenable for high-throughput screenings. Recently, immunoassays based on antibody-antigen specific recognition have played an important role as analytical tools for the quality control of traditional Chinese medicines. Additionally, the indirect competitive enzyme-linked immunosorbent assay (icELISA) is the primary method among immunoassays for analyses of low-molecular-weight natural products [19, 20].

In this study, a specific monoclonal antibody (mAb) against rhein was produced. Based on this mAb, an icELISA method for rhein detection was developed and successfully applied for the determination of rhein in different *Rheum officinale* samples, the results of which were verified by HPLC.

## 2. Materials and Methods

### 2.1. Reagents and Apparatus

Rhein (99% purity) and its analogues used for cross-reactivity studies were obtained from Chengdu Push Biological Technology co., LTD (Chengdu, China). The following reagents for cell fusion and cell culture were purchased from Sigma (St. Louis, MO, USA): polyethylene-glycol (PEG)-2000; Dulbecco's modified Eagle's medium (DMEM); fetal bovine serum (FBS); streptomycin; penicillin; *L*-glutamine; hypoxanthine; aminopterin; and thymidine. Polyoxyethylene sorbitan monolaurate (Tween-20), *o-*phenylenediamine (OPD), and inorganic salts used in the ELISA procedures were obtained from Sinopharm Chemical Reagent Beijing Co. Ltd (Beijing, China). Goat anti-mouse IgG-horseradish peroxidase (IgG-HRP) was obtained from Axxora (Loerrach, Germany). All other reagents and solvents were of analytical grade.

Ninety-six-well polystyrene microtiter plates were purchased from Xiangyushun High Polymer Material Technology Development Tianjin Co., Ltd. (Tianjin, China). Cell culture plates were purchased from Costar (Corning, NY, USA). A direct-heat CO_2_ incubator (311), UV-Vis spectrophotometer (NanoDrop 2000), and microplate reader (Multiskan FC) were purchased from Thermo (Waltham, MA, USA). An electric-heating constant-temperature incubator (ZXDR-2800) was purchased from Shanghai Zhicheng Analytical Instrument Manufacturing Co., Ltd (Shanghai, China). An Agilent 1260 HPLC system was obtained from Agilent (Agilent Technologies, Santa Clara, CA, USA). *Rheum officinale* samples were collected from different regions of China.

### 2.2. Preparation of Immunogen and Coating Antigen

Rhein-BSA and rhein-OVA were produced in our laboratory following the active ester method [21]. In brief, rhein (10 mg), *N*-hydroxysuccinimide (NHS, 6.0 mg), and dicyclohexylcarbodiimide (DCC, 8.0 mg) were dissolved in 2 mL, 100 *μ*L, and 100 *μ*L of absolute *N*,*N*-dimethylformamide (DMF), respectively. NHS was added into rhein solution slowly under constant stirring, and DCC was added subsequently 30 min later. Four hours later, the reaction solution was kept at 4°C for 12 h followed by centrifugation. The BSA/OVA protein solutions (20.0 mg) were dissolved in 2.0 mL of PBS buffer (0.01 M phosphate buffer comprise 0.15 M NaCl, pH 7.5). The result mixture was added into protein solutions dropwise while stirring. The coupling reaction was kept at 4°C overnight. After dialysis with 0.01 M PBS for 72 h, rhein-BSA and rhein-OVA were stored at −40°C for further use.

### 2.3. Production of Monoclonal Antibody

The protocols for immunization, fusion, antibody production, and purification were the same as those described previously [22]. Briefly, five female seven-week-old Bal b/c mice were immunized with the immunogen (rhein-BSA) emulsified in Freund complete adjuvant (FCA) by an intraperitoneal injection for the first exposure. In the following four weeks, two secondary boosters of the same dose (0.2 mg) with incomplete Freund adjuvant (FICA) were administered at regular intervals. The serum of each mouse was collected, and the titer was monitored by the ELISA method five days after the last injection to select the optimum mouse for the subsequent fusion. The best mouse was boost-immunized with immunogen without adjuvants four days before fusion. The antibody-producing splenocytes were fused with SP2/0 myeloma cells by the polyethylene-glycol method. After screening the culture supernatant seven days after fusion, the highest affinity antibody-producing hybridomas were cloned by the limited-dilution method and were selected by icELISA. The clone that had a high antibody titer and good sensitivity in the culture supernatant was expanded. MAb was prepared by the method of introducing ascites into the abdomen, purifying by the ammonium-sulfate precipitation method, followed by dialysis via six changes of distilled water for three days at 4°C and, finally, lyophilization.

### 2.4. Characterization of mAb

Direct ELISA was used to determine the titer of mAb from the ascites fluid. The affinity constant *Kd* of mAb was determined by the icELISA method provided by Beatty [23]. The mAb isotype was determined according to the instructions for the isotype kit from Pierce (Rockford, IL, USA). The specificity of antibody was detected by icELISA, and the IC_50_ (50% inhibition) value was used to calculate the cross-reaction rate with the related compounds.

### 2.5. Procedures for icELISA

There were six steps in the icELISA procedures, and all reactions were carried out at 37°C. First, 96-well microplates were coated with rhein-OVA (100 *μ*L) made in carbonate buffer (0.05 M carbonate buffer, pH 9.6) for 3 h. Second, the plate was washed four times with PBS (0.1-M phosphate buffer containing 0.9% NaCl, pH 7.5), and the unbound sites were blocked with 200 *μ*L of 3% nonfat dry milk in PBS for 30 min. Third, after four washes with PBST (PBS with 0.1% (*v/v*) Tween-20), 50-*μ*L aliquots of various concentrations of the standard diluted in PBSTG (PBST containing 0.5% gelatin, *w/v*) were pipetted into each well, followed by addition of 50 *μ*L of antisera, supernatant, or mAbs diluted in PBSTG. Fourth, the plate was washed with PBST four times after being incubated for 30 min, and then 100-*μ*L goat anti-mouse IgG-HRP conjugate diluted in PBSTG was added to each well. Fifth, the plate was washed four times after being incubated for 30 min. To each well, 100 *μ*L of substrate solution (4 *μ*L of 30% H_2_O_2_ added to 10-mL citrate-phosphate buffer containing 2 mg/mL OPD) was added for color development. Finally, the reaction was stopped with 50 *μ*L of 2 M H_2_SO_4_ after incubating for 10 min. Absorbance was read at 492 nm in the microplate reader. The calibration-curve data were imported into Origin Pro 8.5 (Origin Lab; USA) and fit to a sigmoidal logistical equation. The direct ELISA was almost the same protocol as the icELISA, except that the solutions added in the third step were replaced by 100 *μ*L of mAbs.

### 2.6. Assay Precision and Variation

To determine the accuracy and variation of this icELISA method, the measuring range (0.02 to 1.0 ng/mL) of rhein was measured. For intra-assay (well to well), the range was measured three times in a day, while for interassay (plate to plate and day to day), it was replayed in three consecutive days.

### 2.7. Recovery Experiments

*Rheum officinale* samples were detected by icELISA to calculate the average recovery after different contents of rhein were added. Specifically, the rhein standard was dissolved in absolute methanol and adjusted to a concentration of 50 *μ*g mL^−1^. Different volumes (20, 40, 100, and 200 *μ*L) of rhein stock solutions were spiked into dried *Rheum officinale* powder (5 mg), for which the rhein content was known (3.17 mg g^−1^), and the extract volume was then made up to 1.0 mL with methanol. Spiked samples were extracted under sonication. After four extractions, the supernatant was combined and adjusted to a volume of 10 mL with methanol. The recovery experiment was performed by icELISA after a 20,000-fold dilution. The recovery of spiked rhein was calculated as follows:

(1) $$\begin{array}{c} \text{Recovery}\left(\text{\%}\right)=\;\frac{\mathrm{Measured}\;\mathrm{amount}\text{ of rhein}-\text{ rhein content in unspiked sample}}{\text{spiked amount}}\times 100\%. \end{array}$$

### 2.8. Sample Extraction and Analysis

Seven *Rheum officinale* samples were collected from different regions of China and were powered well. Next, 20 mg of the powdered *Rheum officinale* samples were extracted by 100 mL methanol for 30 min in an ultrasonic bath, which was followed by centrifugation at 8000 rpm for 10 min. Two milliliters of the upper solution were collected and then filtered through a 0.45 *μ*m membrane filter for HPLC analysis. Additionally, the other upper solution was used for rhein detection by icELISA after a 10,000-fold dilution (100 times diluted for each time, repeated twice).

In the HPLC system, a C_18_-column (250 × 4.6 mm) was used as the stationary phase, and the mobile phase consisted of methanol-0.1% phosphoric acid (85 : 15, *v/v*) for the separation. The flow rate was 1.0 mL min^−1^. A 10-*μ*L aliquot of each sample was injected and the detection wavelength of a diode-array detector (DAD) was set as 254 nm.

## 3. Results and Discussion

### 3.1. Preparation of Immunogen and Coating Antigen

The carboxylic acid group in rhein molecule can be conjugated with carrier proteins (BSA/OVA) by active ester system. Therefore, rhein-BSA was prepared as immunogen, while rhein-OVA was prepared as coating antigen for icELISA in this study. Finally, the complete antigen was identified by UV-VIS as Yuan [24] reported, and rhein-BSA and rhein-OVA coupling ratios were 4 : 1 and 3 : 1, respectively.

### 3.2. Characteristics of Monoclonal Antibody

Seven days after fusion, the supernatant of the cell culture medium was screened by icELISA. Four hybridomas of five 96-well plates were strongly positive, and the one with the best sensitivity and selectivity was cloned. After repeated screenings, the clone, 1F8, was expanded and used to produce the antibody. The mAb produced by 1F8 was classified into the IgG1 category, which had a *κ* light chain. The *Kd* of mAb1F8 was 3.8 × 10^−11^ M. Furthermore, the reactivity of mAb against rhein-OVA was tested with various concentrations. The titer (the maximum serum dilution that gave an absorbance of 1.0 in the noncompetitive assay conditions) of the ascites was 1-2 × 10^4^.

### 3.3. Sensitivity

**Figure 1:**
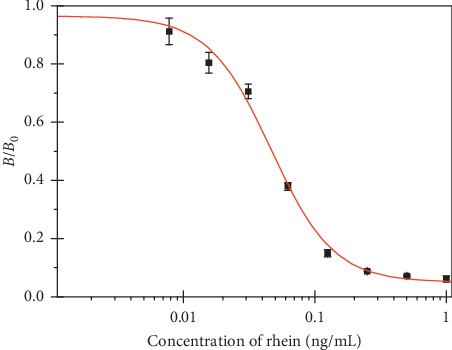
Standard inhibition curve of rhein by icELISA, obtained under optimized conditions. *B*_0_ and *B* were absorbance in the absence and presence of competitors, respectively. Each value represents the mean of three replicates.

The optimal concentrations of the coating antigen, mAb, and IgG-HRP were screened by checkerboard titration. Concentrations of 0.5 *μ*g mL^−1^ rhein-OVA, 0.5 *μ*g mL^−1^ mAb1F8, and 0.2 *μ*g mL^−1^ goat anti-mouse IgG-HRP were selected and used throughout these experiments. A standard inhibition curve for rhein was established by icELISA under optimized conditions (Figure 1). The IC_50_ value and the working range based on 20%–80% of inhibition were 0.05 *μ*g L^−1^ and 0.02–0.11* μ*g L^−1^, respectively. To the best of our knowledge, this is the most sensitive method for rhein detection.

### 3.4. Specificity of the icELISA

Physcion, emodin, rhein, aloe-emodin, and chrysophanol are the major pharmaceutical compounds in *Rheum officinale*. These five anthraquinones have been recorded as quality-control makers of *Rheum officinale* in the China Pharmacopoeia. Some anthrones, such as Sennoside A and Sennoside B, also exist in *Rheum officinale*. Additionally, sennoside A has been recorded as a quality-control marker of *Rheum officinale* in the Korea Pharmacopoeia and the Japan Pharmacopoeia. Rhaponticin, a distyrene derivative, which only exists in fake *Rheum officinale*, has been used for qualitative identification of *Rheum officinale*. Thus, in this study, all the related compounds mentioned above were used to determine and estimate the specificity of mAb1F8 by icELISA and its resultant cross reactivities (CRs).

As shown in Table 1, the mAb revealed high specificity since it only had a relatively high CR with aloe-emodin (27%) and almost no CRs with any of the other compounds (<1%). Compared within the anthraquinones, the CRs of rhein, aloe-emodin, emodin, chrysophanol, and physcion were decreased. In general, the epitopes distant from the coupling site (for rhein, it was –COOH that we used here) were more inclined to be recognized by antibodies, whereas epitopes neighboring the conjugation site tended to be less well-recognized [25]. The mAb1F8 had certain recognition ability to aloe-emodin, and this was mainly due to the similar chemical structure between rhein and aloe-emodin. The only difference between these structures is that the carboxyl (–COOH) of rhein is replaced by hydroxymethyl (–CH_2_OH).

Aside from the mAb1F8 that we developed in this study, Zhang [26] produced an antibody against rhein from eggs of immunized female roman chickens with rhein-BSA. The egg-yolk antibody had a high CR with rheum emodin (64.58%), while the CRs with aloe-emodin, physcion, and chrysophanol were unanalyzed.

### 3.5. Assay Precision and Variation

Intra-assay and interassay precision were studied. From the result of Table 2, the maximum coefficient of RSD intra-assay was 4.30%, while that of interassay was 6.84%. The RSDs of interassay were higher than that of intra-assay in average.

### 3.6. Recovery Experiments

As shown in Table 3, the recovery experiment showed good recoveries of rhein from *Rheum officinale* sample solutions, which ranged from 84.19% to 102.90%, while the RSD ranged from 1.00% to 6.96%.

### 3.7. Comparison of ELISA and HPLC Determination of Rhein in Different *Rheum Officinale* Samples

**Figure 2:**
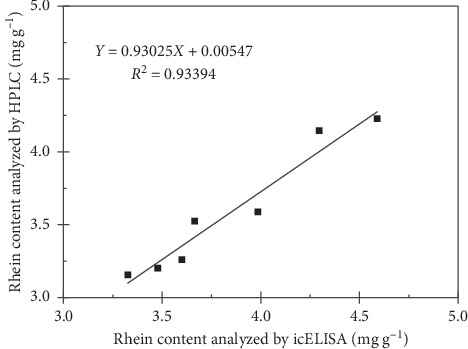
Correlation between rhein content of *Rheum officinale* samples determined by icELISA and by HPLC.

The rhein contents of seven *Rheum officinale* samples were detected by icELISA and HPLC. For HPLC analysis, the calibration curve of rhein showed good linearity and was as follows: *Y* = 2718.3*X* + 2.5306, *R*^*2*^ = 0.9998, where *Y* is the peak area of rhein and *X* is the concentration (*μ*g mL^−1^) of rhein. As shown in Table 4, the rhein contents ranged from 3.15 to 4.23 mg g^−1^. The results obtained from icELISA (*X*, mg g^−1^) were quite similar to those of the HPLC (*Y*, mg g^−1^) method, and they showed a high correlation (*R*^2^ = 0.93394) with the linear regression equation of *Y* = 0.93025*X* + 0.00547 (Figure 2). Taken together, these results suggest that our developed icELISA could be used as an effective and accurate method for rhein analysis.

## 4. Conclusions

To our knowledge, this is the first and most specific mAb against rhein, which has been produced and applied to an icELISA for rhein determination in *Rheum officinale* samples. Additionally, the icELISA was highly sensitive to rhein, with a corresponding IC_50_ value of 0.05 *μ*g L^−1^. The results obtained from the icELISA corroborated results from the HPLC analysis. Taken together, our developed icELISA is suitable for rhein analysis; this method is simple, rapid, cost-effective, high-throughput, and could be an important tool for the quality control of *Rheum officinale*.

## Figures and Tables

**Table 1:** Cross reactivities of mAb1F8 against various compounds.

Compound	Structure	IC_50_ (*μ*g L^−1^)	CR (%)
Rhein	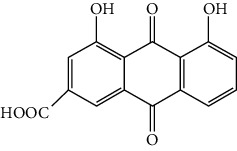	0.044	100
Aloe-emodin	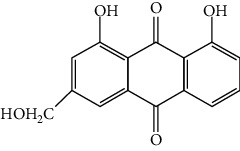	0.162	27
Emodin	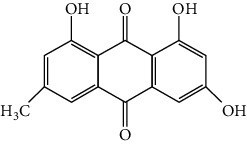	4.946	0.9
Chrysophanol	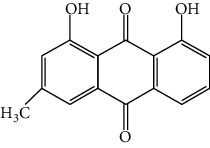	14.144	0.3
Physcion	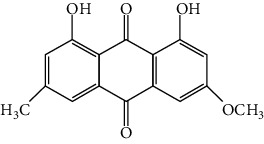	50.710	0.1
Sennoside A	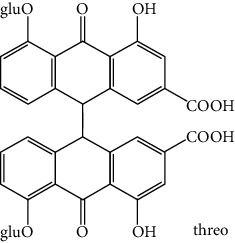	1393.901	0.005
Sennoside B	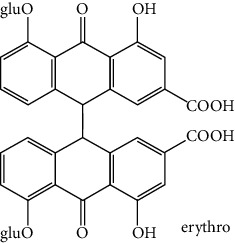	—^a^	<0.001
Rhaponticin	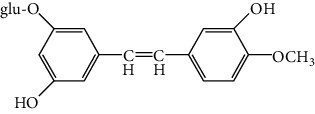	—	<0.001

^a^No inhibition was observed at up to 20,000 *μ*g L^−1^ of the compound.

**Table 2:** Validations among ELISA runs for the analysis of rhein.

Rhein (ng/mL)	RSD (%)	
	Intra-assay	Interassay
0	3.41	6.84
1.00	0.38	0.64
0.50	1.31	0.70
0.25	0.65	0.86
0.16	2.73	3.59
0.06	1.92	3.87
0.03	4.30	4.20
0.02	2.13	3.53

**Table 3:** Recovery of rhein determined by icELISA in spiked samples.

Spiked level (*μ*g)	Mean amount^a^ (*μ*g)	Recovery^b^ (%)
0	3.17 ± 0.29^c^	0
1	4.06 ± 0.05	89.17 ± 4.56
2	5.38 ± 0.23	102.90 ± 6.96
5	7.38 ± 0.29	84.19 ± 5.72
10	13.15 ± 0.10	99.77 ± 1.00

^a^Data were mean ± SD from triplicate samples for each level. ^b^The percentage of recovery was calculated as follows: recovery (%) = (measured amount − control)/spiked amount × 100. ^c^The zero spiked level was obtained by the HPLC method and used as a control.

**Table 4:** Determination of rhein contents in *Rheum officinale* samples by icELISA and HPLC.

Samples^a^	Concentrations of rhein (mg g^−1^)	
	icELISA	HPLC
Gansu	3.99 ± 0.20^b^	3.59 ± 0.08
Anguo, Hebei	3.66 ± 0.22	3.52 ± 0.01
Bozhou, Anhui	3.33 ± 0.19	3.15 ± 0.09
Bozhou, Anhui	3.48 ± 0.29	3.20 ± 0.03
Chengdu, Sichuan	4.30 ± 0.29	4.14 ± 0.02
Chengdu, Sichuan	3.60 ± 0.35	3.26 ± 0.01
Ganzhi, Sichuan	4.59 ± 0.36	4.23 ± 0.04

^a^Each sample was analyzed in triplicate. ^b^The data represented the mean ± SD.

## Data Availability

The data used to support the findings of this study are available from the corresponding author upon request.

## Supplementary Materials

Supplementary Materials Graphical abstract of this article: for the quality control of rhubarb, an indirect competitive enzyme-linked immunosorbent assay (icELISA) for rhein (one of the quality control marker) detection was developed. Also, the icELISA was verified by HPLC.
