# Supplementary figures and images for: Development of an Enzyme-Linked Immunosorbent Assay Method for the Detection of Rhein in Rheum officinale
Source: Int J Anal Chem. 2020 Mar 16;2020:4294826. doi: 10.1155/2020/4294826 (PMC7102413; doi:10.1155/2020/4294826)

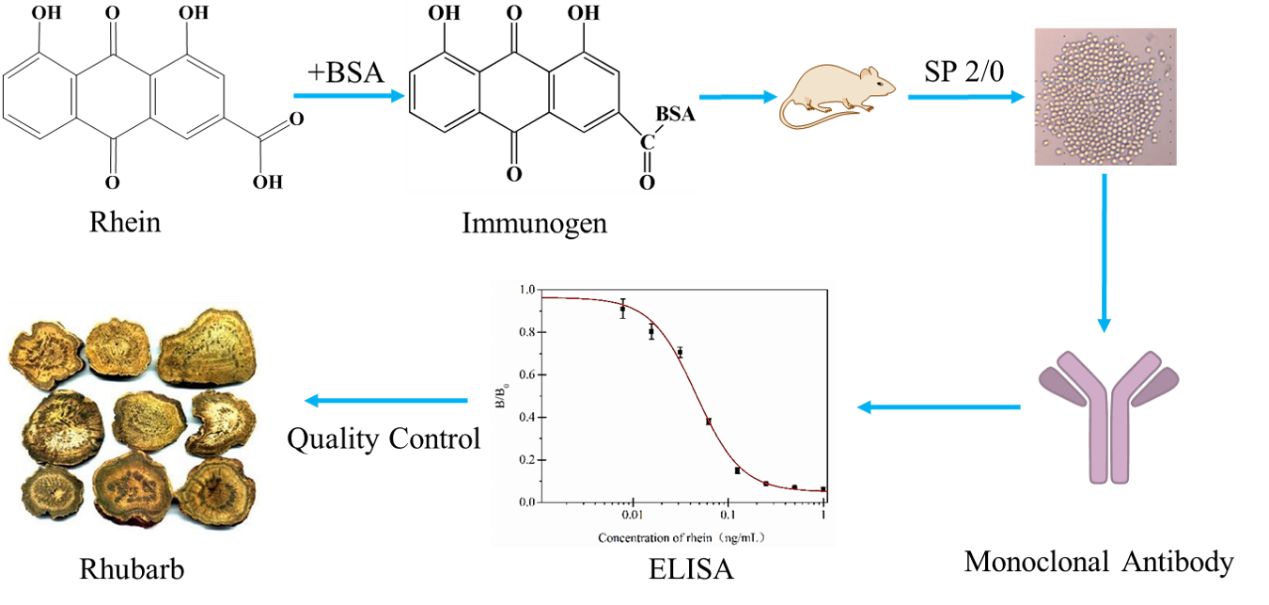

Supplement: Supplementary Materials — Graphical abstract of this article: for the quality control of rhubarb, an indirect competitive enzyme-linked immunosorbent assay (icELISA) for rhein (one of the quality control marker) detection was developed. Also, the icELISA was verified by HPLC. [file 4294826.f1.docx]
